# Adverse childhood experiences, resilience, and syringe services program attendance among persons who inject drugs in Northeast Georgia, USA: A mediation analysis

**DOI:** 10.1016/j.dadr.2024.100309

**Published:** 2024-12-10

**Authors:** Mohammad Rifat Haider, Samantha Clinton, Monique J. Brown, Nathan B. Hansen

**Affiliations:** a Department of Health Policy and Management, College of Public Health, University of Georgia, Athens, GA, United States; b Department of Health Promotion and Behavior, College of Public Health, University of Georgia, Athens, GA, United States; c Department of Epidemiology and Biostatistics, Arnold School of Public Health, University of South Carolina, Columbia, SC, United States; d South Carolina SmartState Center for Healthcare Quality, Arnold School of Public Health, University of South Carolina, Columbia, SC, United States; e Rural and Minority Health Research Center, Arnold School of Public Health, University of South Carolina, Columbia, SC, United States; f Office for the Study on Aging, Arnold School of Public Health, University of South Carolina, Columbia, SC, United States; g Centre for Health Systems Research & Development, University of the Free State, Bloemfontein, South Africa

**Keywords:** Adverse childhood experiences, Resilience, Syringe services program, People who inject drugs

## Abstract

**Background:**

Syringe services programs (SSP) are evidence-based venues offering harm reduction services to persons who inject drugs (PWID), such as sterile syringes, STI/HIV testing, and linkage to care to decrease drug use-related morbidities and mortalities. Adverse childhood experiences (ACEs) have been linked with reduced resilience, while increased resilience can help PWID attend SSPs. This study examined the potential mediating role of resilience between ACEs and SSP attendance among PWID.

**Methods:**

Data were collected from adult HIV-negative PWID in northeast Georgia, between February-December 2023 (N = 173). Data were collected on SSP attendance (Yes vs. No), resilience, and ACEs. Covariates included age, gender, sexual orientation, race/ethnicity, education, homelessness, HIV risk behavior, syringe sharing, syringe use frequency, and primary drug. Path analysis was performed using Stata 18.0.

**Results:**

The majority of PWID were cisgender men (68.8 %), heterosexual (92.5 %), homeless (93.6 %), had HIV risk behavior (65.9 %), had high resilience (54.3 %), and had never attended SSP (64.2 %). The mean number of ACEs was 4.1 (SD=3.2). After adjusting for covariates, high resilience was positively associated with SSP attendance (β= 0.204; p = 0.005). ACEs were negatively associated with high resilience (β= −0.035 p = 0.005) and SSP attendance (β= −0.026; p = 0.034). ACEs had a significant indirect effect on SSP attendance through high resilience (β= −0.007; p = 0.044).

**Conclusions:**

Results indicate that resilience may mediate the relationship between ACEs and SSP attendance among PWID. It is important to develop and implement trauma-informed and resilience-based interventions to address the mental and sexual health challenges of PWID with a history of ACEs.

## Highlights

• Most Persons who inject drugs (PWID) reported ≥ 4 mean Adverse childhood experiences (ACEs), and had high resilience.
• ACEs were negatively associated with high resilience and syringe services program (SSP) attendance among PWID.
• High resilience was positively associated with SSP attendance among PWID.
• Resilience mediated the relationship between ACEs and SSP attendance among PWID.

## 1 Introduction

Globally, approximately 14.8 million people aged 15–64 inject drugs, and around 3.1 million of them live in the United States (US) (Degenhardt et al., 2023). Syringe services programs (SSPs) are one of the evidence-based harm reduction interventions recommended by the World Health Organization (WHO) for preventing the transmission of HIV and hepatitis C virus (HCV) (Nelson, 2023). However, due to the federal ban on funding SSPs from 1988 to 2016, the number of SSPs was far from ideal in the US (Des Jarlais et al., 2020). Moreover, SSP attendance is affected by low community acceptance and persistent legal, policy, and funding barriers (Jones, 2019).

While the Southern states do not have the highest rates of drug usage in the US, the South does have the highest incidence of HIV morbidity and mortality in the US (AIDSVu, 2023, CDC, 2016, Philbin et al., 2019). Specifically within Atlanta, Georgia’s capital, injection drug use accounts for approximately six to seven percent of all HIV cases (AIDSVu, 2020). Surveys of people who inject drugs (PWID) have shown that PWID are generally aware of the risks related to injection drug use (IDU), including HIV, HCV, and overdose. Ohringer et al. (2021) found that three-quarters of their PWID sample were aware of the risk of HCV acquisition, while another study reported that all PWID surveyed were aware of their HIV/HCV acquisition risk (Sirikantraporn et al., 2012). Among a sample of opioid users, nearly 61 % had experienced an overdose and 75 % had witnessed an overdose (Kestler et al., 2017). Simply being aware of the IDU risks, however, is only one step towards the uptake of preventative care. Awareness of, and access to preventative care is further compounded by the lack of access to healthcare, demonstrated by the significantly higher uninsured rate in Georgia when compared to the national average (Terlizzi and Cohen, 2023).

The majority of the US SSPs are located in urban and suburban areas, predominantly within the North and along the Western coast (Canary et al., 2017; Facente et al., 2024; North American Syringe Exchange Network (NASEN, 2024). Georgia is located deep within the Southern US, where SSPs are less commonly found (North American Syringe Exchange Network (NASEN, 2024). SSPs can provide access to harm reduction provisions, like sterile syringes, HIV/STI testing, pre-exposure prophylaxis (PrEP), and overdose reversal resources, e.g., naloxone (CDC, 2024). Access to, and utilization of SSPs to obtain sterile equipment has been demonstrated to reduce the probability of HIV risk behaviors such as syringe sharing (Sirikantraporn et al., 2012). Moreover, SSPs serve to fill the gap in care for uninsured PWID (Philbin et al., 2019). Notable use of SSPs among PWID has been documented, with one study citing approximately half of their rural sample ever having used an SSP and another reporting nearly 44 % of their sample having used an SSP in the previous 30 days (Ballard et al., 2023, Lancaster et al., 2020). SSP providers also note an increase in the use of their services, especially for naloxone in the wake of high drug overdose rates during COVID-19 (Frost et al., 2022). However, barriers that limit SSP utilization, such as stigma and a lack of awareness, continue to exist (Lancaster et al., 2020).

The use of health promoting services has been found to be impacted by traumatic life events such as adverse childhood experiences (ACEs) (Randhawa et al., 2018). ACEs are commonly reported among PWID (Bryant et al., 2020, Hughes et al., 2019, Leza et al., 2021). ACEs are associated with increased risk-taking as well as an increased likelihood of adverse health outcomes, like substance use disorder, later in life (Borgert et al., 2022, CDC, 2019, Hughes et al., 2019). Individuals who have experienced an ACE tend to surround themselves with individuals who engage in risky behaviors, further perpetuating the cycle of negative outcomes (Trinidad, 2021). Studies have found that among individuals with substance use disorders who had experienced ACEs, decreased interactions with healthcare services and increased withdrawal from treatment programs were reported (Kang et al., 2002, Randhawa et al., 2018). Limited research exists on how ACEs impact one’s use of SSPs, however, given that having experienced ACEs is linked with lower access to healthcare, indicating a possible negative impact on the use of harm reduction services (Randhawa et al., 2018). Conversely, factors such as resilience and empowerment among PWID can aid in healthcare seeking behaviors (Surratt et al., 2022).

Research suggests that resilience, or “the process and outcome of successfully adapting to difficult or challenging life experiences,” mediates the relationship between ACEs and substance misuse (APA, 2018, Wang et al., 2021). Resilience helps people identify and utilize coping resources and enables them to rise over adversity (Aburn et al., 2016). Among marginalized populations, resilience has been found to be a protective factor for a number of health outcomes (Huang, 2024, Rivas-Koehl et al., 2022). Lower levels of resilience are associated with both a higher number of ACEs and an increased risk of substance use (Kendler et al., 2021, Wang et al., 2021). Conversely, resilience can be found in many forms, such as supportive childhood relationships and continuous adult support in childhood; these relationships are associated with a decrease in risky behaviors, such as substance use (Bellis et al., 2017, Hughes et al., 2019). Limited research exists regarding the role resilience plays in SSP utilization, specifically among individuals who have experienced ACEs. Resilience has been demonstrated, however, in the form of planning ahead to have sterile equipment ready for use, therefore decreasing the odds of performing a risky behavior such as syringe sharing (Sirikantraporn et al., 2012). This research suggests that resilience can potentially help mitigate risky injection practices, especially with the assistance of SSPs.

PWID may experience challenges in life, one such example being experiencing an increased number of ACEs (Leza et al., 2021). Experiencing ACEs has been linked to the outcome of substance use in adulthood, which is further complicated by the fact that those who have experienced ACEs are less likely to interact with health services (CDC, 2019, Testa et al., 2022). Resilience has been shown to be protective against negative experiences; however, further research should be done to investigate its role in harm reduction among PWID (Huang, 2024). While SSP attendance, ACEs, and resilience have been studied individually, there is a fundamental lack of research on the interaction of these variables. This study aims to investigate the mediational role resilience plays in SSP attendance among PWID exposed to ACEs. We hypothesize that having high resilience will increase the likelihood of ever having used an SSP, even in the presence of ACEs. Based on our findings, we hope that facilities that work with PWID will be able to create tailored interventions focused on building resilience and lend increased support to the utilization of SSPs.

## 2 Material and methods

### 2.1 Human subjects

Participants were recruited based on these eligibility criteria: (1) ≥ 18 years old; (2) currently injecting drugs (within last 30 days); (3) shows injection marks (Deren et al., 2017); (4) never tested positive for HIV; (5) understands, speaks, writes English; and (6) willing to participate in a 35–40-minute survey. We excluded PWID living with HIV because one of the aims of the broader study was to assess awareness and use of PrEP among HIV-negative PWID. This study was reviewed and approved by the University of Georgia Institutional Review Board.

### 2.2 Study setting and design

This cross-sectional study was conducted during February-December 2023 in Athens, Georgia. This study was conducted in a predominately rural area of Georgia, with limited harm reduction service locations. We collaborated with two human services organizations in Athens, which provide services to marginalized populations living in Northeast Georgia. Both of these organizations provide services aimed primarily at those experiencing or are at risk of homelessness. Amenities such as food, showers, clothing, laundry, internet access, and educational classes are offered. We recruited the participants while they attended services at these organizations. Study participants were recruited using convenience sampling.

### 2.3 Data collection and procedures

Organization staff informed potential participants about the survey opportunity and directed those interested to the study team. The study team administered an electronic eligibility screening tool, then, with help from the organization staff who had lived experiences of IDU, checked for physical evidence of the participants’ recent injection use. After confirming eligibility, participants consented and were then enrolled as eligible PWID in the study. After providing written consent, the individual completed the survey. For their participation, each PWID received a $30 gift card. Responses were collected and stored in Qualtrics.

### 2.4 Measures

#### 2.4.1 SSP attendance

The outcome variable, SSP attendance, was determined from the question, “Have you ever used syringe services programs for syringe exchange?” (Yes vs. No).

#### 2.4.2 Adverse childhood experiences (ACEs)

The exposure variable, ACEs, was obtained using the 11-item ACEs scale adapted from the Centers for Disease Control and Prevention–Kaiser ACE Study (CDC, 2021). The ACE questions capture adverse experiences in their first 18 years of life covering traumatic experiences (emotional, physical, sexual), household challenges (mother treated violently, substance use issues in the household, mental illness in the household, parental separation/divorce, incarcerated household member), and neglect (emotional, physical). The questions with yes/no responses were coded (0 “No” and 1 “Yes”); for questions with responses with frequency of occurrence (never, once, and more than once), we recoded them into (0 “Never”, and 1 “At least once”). Responses were summed into a continuous variable (range 0–11) for statistical analysis.

#### 2.4.3 Resilience

We used the Connor-Davidson Resilience Scale-10 (CD-RISC-10) to measure resilience. The 10-item validated scale asks about how one handles a stressful situation with Likert scale responses (0 “not at all”, 1 “a little bit”, 2 “moderately”, 3 “quite a bit”, and 4 “extremely”). We summed all items to obtain a resilience score (range 0–40). We then categorized study participants into low resilience (≤ 25.5) and high resilience (> 25.5) groups (Campbell-Sills and Stein, 2007).

#### 2.4.4 Covariates

Among PWID, factors including being White, older in age, cisgender male, heterosexual, being of lower education, experiences of homelessness, participating in HIV-risk behaviors, and use of certain drugs have been found to be associated with impacts on one’s ability to access medical services (Randhawa et al., 2018). Experiences of ACEs or other adverse mental health outcomes among both PWID and the general population are also associated with increased HIV-related risk behaviors, including risky injection behaviors such as syringe sharing, and being cisgender male (Dutra et al., 2024, Fang et al., 2016, Mackesy-Amiti et al., 2014). Associations have also been observed between increased ACEs and having been tested for HIV (Dutra et al., 2024). Due to these established associations, the following covariates were selected in order to minimize the concern of confounding in the investigation of the relationship between ACEs, SSP use, and resilience.

Covariates included age (25–34, 35–44, 45–54, ≥ 55), gender (transgender, cisgender woman, cisgender man), sexual orientation (lesbian/gay/bisexual, heterosexual), race/ethnicity (Non-Hispanic White, Non-Hispanic Black, Non-Hispanic other, Hispanic), education (less than high school, high school, college or higher), currently homeless (yes, no), HIV risk self-perception (yes, no), HIV risk behavior over the previous 12 months (condomless vaginal or anal sex, transactional sex, group sex, or sex under the influence of drugs or alcohol), syringe sharing over the previous 4 weeks (yes, no), syringe use frequency (1 time, ≥2 times), and primary drug (cocaine, heroin, fentanyl, other opioids, methamphetamine).

#### 2.4.5 Statistical analysis

Sample characteristics were calculated, and bivariate analysis was conducted. For the continuous exposure variable (ACEs), mean and standard deviation were calculated. Frequency and percentage for the binary outcome variable (SSP attendance) were determined. We conducted t-tests for binary covariates and F-tests for covariates with multiple categories to test for statistically significant differences in ACEs. We also conducted the chi-square test to find out the differences in the proportion of PWID who ever attended SSP.

We conducted path analysis to determine the association between experiencing ACEs (exposure), resilience (mediator), and SSP attendance (outcome), both crude and adjusted for sociodemographic characteristics. Path analyses allow for the investigation of a mediating variable on the relationship between an exposure variable and an outcome variable. We obtained direct and indirect standardized adjusted estimates between ACEs, resilience, and SSP participation. Indirect effects model the pathways between the exposure and outcome variable, accounting for the impact of the mediating variable, while the direct effects demonstrate the relationships between variables without accounting for a mediating variable (Richiardi et al., 2013). We considered p-value < 0.05 as statistically significant for all analyses. All analyses were performed using Stata 18.0.

## 3 Results

Table 1 shows the distribution of sociodemographic characteristics, mean and standard deviation for ACEs, and frequency and proportion for resilience. Out of the 186 PWID who started the survey, 182 completed the survey, and 173 had complete information for the study variables and were included in this study. A little over one-third (35.8 %) of PWID ever attended SSP. The majority of PWID were cisgender men (68.8 %), heterosexual (92.5 %), non-Hispanic Black (48.6 %), currently homeless (93.6 %), perceived less lifetime risk for HIV transmission (53.8 %), had HIV risk behavior during the previous 12 months (65.9 %), had used one syringe multiple times (61.3 %), and had high resilience (54.3 %). The mean number of ACEs experienced was 4.1 (SD=3.2). There was a statistically significant difference in SSP attendance by resilience, when low and high resilience categories were compared (p = 0.003). A statistically significant difference was also seen in SSP attendance by a respondent’s primary drug use, with more PWID used methamphetamine attending SSP than PWID used other drug (p = 0.004).

**Table 1:** Distribution of sociodemographic characteristics, adverse childhood experiences (ACEs), and syringe services program (SSP) attendance among persons who inject drugs (PWID) , (N = 173).

**Characteristics**	**n (%)**	**ACEs** **Mean (SD)**	**p-Value**	**SSP Attendance** **n (%)**	**No SSP Attendance** **n (%)**	**p-Value**
**Overall**		4.1 (3.2)		62 (35.8)	111 (64.2)	
**Age**			**0.005**			0.257
25−34	26 (15.0)	5.3 (3.2)		12 (19.4)	14 (12.6)	
35−44	49 (28.3)	4.7 (3.1)		17 (27.4)	32 (28.8)	
45−54	54 (31.2)	4.0 (3.3)		22 (35.5)	32 (28.8)	
≥ 55	44 (25.4)	2.8 (2.9)		11 (17.7)	33 (29.7)	
**Gender**			0.158			0.365
Transgender	3 (1.7)	6.7 (4.5)		0 (0.0)	3 (2.7)	
Cisgender Woman	51 (29.5)	4.5 (3.3)		17 (27.4)	34 (30.6)	
Cisgender Man	119 (68.8)	3.8 (3.1)		45 (72.6)	74 (66.7)	
**Sexual Orientation**			**0.011**			0.692
Heterosexual	160 (92.5)	3.9 (3.1)		58 (93.6)	102 (91.9)	
Lesbian, Gay, Bisexual	13 (7.5)	6.2 (3.3)		4 (6.4)	9 (8.1)	
**Race/Ethnicity**			**0.006**			0.371
Non-Hispanic White	68 (37.3)	4.9 (3.1)		29 (46.8)	39 (35.1)	
Non-Hispanic Black	84 (48.6)	3.2 (3.0)		25 (40.3)	59 (53.2)	
Non-Hispanic Other	4 (2.3)	3.5 (3.7)		1 (1.6)	3 (2.7)	
Hispanic	17 (9.8)	5.0 (3.5)		7 (11.3)	10 (9.0)	
**Education**			0.122			0.630
Less than High School	61 (35.3)	4.6 (3.4)		19 (30.6)	42 (37.9)	
High School	72 (41.6)	3.5 (2.7)		28 (45.2)	44 (39.6)	
College or higher	40 (23.1)	4.4 (3.6)		15 (24.2)	25 (22.5)	
**Currently Homeless**			0.181			0.540
No	11 (6.4)	2.8 (3.4)		3 (4.8)	8 (7.2)	
Yes	162 (93.6)	4.2 (3.2)		59 (95.2)	103 (92.8)	
**HIV Risk Self-Perception**			0.381			0.672
No	93 (53.8)	3.9 (3.1)		32 (51.6)	61 (55.0)	
Yes	80 (46.2)	4.3 (3.6)		30 (48.4)	50 (45.0)	
**HIV Risk Behaviors in the Previous 12 Months**			**0.035**			0.702
No	59 (34.1)	3.4 (3.4)		20 (32.3)	39 (35.1)	
Yes	114 (65.9)	4.4 (3.0)		42 (67.7)	72 (64.9)	
**Needle Sharing**			**0.025**			0.717
No	117 (67.6)	3.7 (3.2)		43 (69.3)	74 (66.7)	
Yes	56 (32.4)	4.9 (3.0)		19 (30.7)	37 (33.3)	
**Needle Use Frequency**			0.195			0.327
1 time	67 (38.7)	3.7 (3.1)		21 (33.9)	46 (41.4)	
2 + times	106 (61.3)	4.3 (3.2)		41 (66.1)	65 (58.6)	
**Primary Drug**			0.118			**0.004**
Cocaine	35 (20.2)	3.0 (3.1)		11 (17.7)	24 (21.6)	
Heroin	34 (19.7)	4.1 (3.0)		5 (8.1)	29 (26.1)	
Fentanyl	16 (9.2)	3.4 (2.7)		9 (14.5)	7 (6.3)	
Other opioid	9 (5.2)	5.3 (3.6)		1 (1.6)	8 (7.2)	
Methamphetamine	79 (45.7)	4.5 (3.3)		36 (58.1)	43 (38.7)	
**Resilience**			**0.002**			**0.003**
Low	79 (45.7)	4.9 (3.2)		19 (30.7)	60 (54.0)	
High	94 (54.3)	3.4 (3.0)		43 (69.3)	51 (46.0)	

**Bolded** p-values are statistically significant at p < 0.05.

**Fig. 1:**
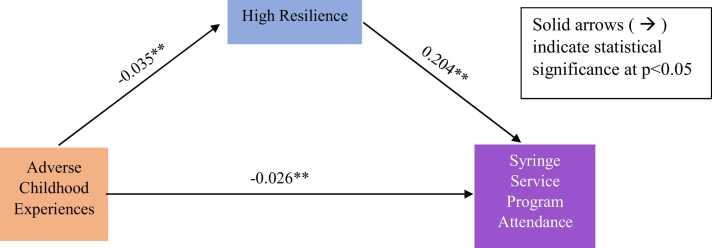
Mediating Pathway between Adverse Childhood Experiences (ACEs), Resilience, Syringe Services Program (SSP) Attendance among Persons Who Inject Drugs (PWID). *Note.* **p < 0.01. Adjusted effect estimates are controlled for age, gender, sexual orientation, race/ethnicity, education, homelessness, HIV risk perception, HIV sexual risk behavior (having any condomless vaginal and anal sex, transactional sex with money or drugs, group sex, or sex under the influence of drugs or alcohol), risky injection behavior (reporting sharing of used needles and using the same needle for more than one time for injection drug use), needle use frequency, primary drug used.

Fig. 1 shows the direct standardized estimates between ACEs, resilience, and SSP attendance. After adjusting for the covariates, the direct effect of ACEs was negatively associated with SSP attendance (β=-0.034; p = 0.004). Resilience was directly and positively associated with SSP attendance (β=0.204; p = 0.005) and ACEs were directly and negatively associated with resilience (β=-0.035; p = 0.004). Resilience significantly mediated the association between ACEs and SSP attendance as an indirect effect (β=-0.007; p = 0.044).

## 4 Discussion

We found a high mean ACEs score among PWID, which aligns with the existing literature (Leza et al., 2021). Previous studies have found that experiencing a higher number of ACEs generally is correlated with an increased risk of substance use disorder in adulthood (Bryant et al., 2020, Leza et al., 2021). Our results also showed that our sample of PWID had high resilience, which is in agreement with other findings among rural participants (Surratt et al., 2022). Research has demonstrated that PWID have expressed their resilience via increased health management, indicating that developing and providing interventions teaching skills to increase resilience could lead to increased SSP use (Surratt et al., 2022).

We found that there is a negative association between ACEs and SSP attendance. Other studies also found a negative relation between ACEs and uptake of health-promoting behaviors, like cancer screening, HIV testing, and COVID-19 vaccination (Brandford et al., 2023, Dyer et al., 2022, Schnarrs et al., 2022). The link between ACEs and substance use is well-established (Broekhof et al., 2023, Cuomo et al., 2008, Kendler et al., 2000, McLaughlin et al., 2010); we add to this that ACEs are negatively associated with the uptake of harm reduction services among PWID. Childhood trauma can exert unique detrimental effects on an individual’s emotional, behavioral, cognitive, and social development through altering the early development of pleasure and reward centers, as well as emotion regulation and impulse control areas in the brain (Herzog and Schmahl, 2018, Sheridan and McLaughlin, 2014), which might contribute to opting for adverse behaviors like substance use over opting for harm reduction activities. Future interventions among PWID could utilize peer education techniques to increase SSP awareness and attendance. PWID have demonstrated an increased willingness to attend SSPs when vouched for by peers (Surratt et al., 2022). Peer education programs have also been shown to decrease risky injection behaviors (Garfein et al., 2007). These techniques could potentially aid in overcoming barriers associated with ACEs when accessing SSPs, however, further research should be done in this area.

We found that resilience mitigates the effect of ACEs on SSP attendance. Other studies also found that resilience was protective among individuals with substance use disorder and ACEs (Bellis et al., 2017, Fisher et al., 2020, He et al., 2022). Resilience is a dynamic process of adapting to stress and traumatic events (Luthar et al., 2000). Resilience can be fostered through harnessing inner resources, like coping skills and self-esteem; and community resources, like family and friendships (Marriott et al., 2014, Panter-Brick, 2014). Resilience-based interventions have been effective in mitigating childhood trauma (Biancarelli et al., 2019, Chandler et al., 2015) and may improve harm reduction services uptake among PWID. SSPs can also foster an individual’s resilience by allowing PWID to manage their health through HIV/STI testing and reducing barriers to care, such as stigma, thereby increasing the likelihood of an individual to return (Surratt et al., 2022).

The study findings suggest that developing trauma-informed and resilience-based interventions to address harm reduction services uptake among PWID with ACEs is a valuable area for research. Creating interventions focused on addressing ACEs and building resilience has the ability to increase SSP attendance by breaking down barriers and promoting facilitators to utilize harm reduction services. Since SSPs are hubs for providing harm reduction services among PWID, increasing utilization can have multiple benefits such as reducing HIV/HCV and drug overdose mortality and morbidity through testing, education, resources, and linkage to care. Future research may assess alternative mediators between ACEs and SSP attendance, such as depressive symptoms and post-traumatic stress disorder.

### 4.1 Strengths and limitations

Some limitations of the study warrant discussion. First, the cross-sectional nature of the data does not allow for establishing the temporal sequence between the mediator and outcome. However, ACEs occurred before 18 years, thus a temporal sequence can be established between ACEs and SSP attendance, which may be mediated by resilience. While individuals with higher resilience may be more likely to seek services, it is also possible that the nature of SSP services may increase one’s resilience. Second, the respondents might experience recall or social desirability biases when responding to ACEs questions, which may result in under-reporting of ACEs. Third, a convenience sample of PWID may not be entirely representative of the population. However, to our best knowledge, few studies have been conducted among PWID living in the Deep South, and we provide valuable insight into the mediating role of resilience between ACEs and SSP attendance among PWID in this region. Fourth, participants were only asked if they had ever attended an SSP and not asked how many times or if they attended regularly. This information could potentially alter the relationships demonstrated.

## 5 Conclusion

In conclusion, we found that this population of PWID had experienced a large number of ACEs, and SSP attendance was lower among those with a greater number of ACEs. However, many participants also reported high levels of resilience. Our path model found that high resilience may mediate the relationship between ACEs and SSP attendance among PWID. Due to these findings, PWID reporting a large number of ACEs should receive interventions focused on increasing resilience in an effort to increase SSP attendance.

## Ethics

Ethics approval for this study was obtained from the University of Georgia (UGA) Institutional Review Board (Project00005456).

## Role of funding source

The content is solely the responsibility of the authors and does not necessarily represent the official views of the National Institutes of Health. We would also like to acknowledge the important contributions of our study participants and the community partners.

## Funding

This project was supported by the University of Georgia (10.13039/100007699UGA) Faculty Seed Grants in Sciences and Engineering FY2023. Mohammad Rifat Haider is supported by grant K01DA059329 from the National Institute on Drug Abuse (10.13039/100000026NIDA). Monique J. Brown is supported by grant K01MH115794 from the National Institute of Mental Health (10.13039/100000025NIMH). Nathan B. Hansen is supported by the grants R01HD092185 from the Eunice Kennedy Shriver National Institute of Child Health and Human Development (10.13039/100000071NICHD) and R34MH13256 from NIMH.

## Author disclosure

All authors have contributed to and approved the final manuscript.

## CRediT authorship contribution statement

**Mohammad Rifat Haider:** Writing – review & editing, Writing – original draft, Investigation, Funding acquisition, Formal analysis, Data curation, Conceptualization. **Samantha Clinton:** Writing – review & editing, Writing – original draft. **Monique J. Brown:** Writing – review & editing. **Nathan B. Hansen:** Writing – review & editing, Resources, Conceptualization.

## Declaration of Competing Interest

The authors have no conflict of interest to declare.

## Data Availability

The datasets generated and/or analyzed during the current study are not publicly available due to the highly sensitive nature of the data but may be available from the corresponding author upon reasonable request.
